# Protein Kinase Inhibitor-Mediated Immunoprophylactic and Immunotherapeutic Control of Colon Cancer

**DOI:** 10.3389/fimmu.2022.875764

**Published:** 2022-04-28

**Authors:** Silvia Ghione, Cindy Racoeur, Nesrine Mabrouk, Jingxuan Shan, Emma Groetz, Elise Ballot, Caroline Truntzer, Lotfi Chouchane, Frédérique Végran, Catherine Paul, Stéphanie Plenchette, Ali Bettaieb

**Affiliations:** ^1^Laboratoire d’Immunologie et Immunothérapie des Cancers (LIIC), EA7269, Université Bourgogne Franche-Comté, Dijon, France; ^2^LIIC, Ecole Pratique des Hautes Etudes (EPHE), Paris Sciences et Lettres (PSL) Research University, Paris, France; ^3^Genetic Intelligence Laboratory, Weill Cornell Medicine-Qatar, Qatar Foundation, Doha, Qatar; ^4^Plateforme de Transfert en Biologie Cancérologique, Centre Georges François Leclerc, Dijon, France; ^5^Team CAdIR, Institut National de la Santé et de la Recherche Médicale (INSERM) U1231, Lipids, Nutrition and Cancer, Dijon, France; ^6^University of Burgundy and Franche-Comté, Dijon, France

**Keywords:** H89, kinase inhibitor, colorectal cancer, immunotherapy, immunoprophylaxis

## Abstract

Immunotherapy has allowed major advances in oncology in the past years, in particular with the development of immune checkpoint inhibitors, but the clinical benefits are still limited, particularly in colorectal cancer (CRC). Our scientific approach is based on the search for innovative immunotherapy with a final goal that aims to induce an effective antitumor immune response in CRC. Here, we focused on a multikinase inhibitor, H89. We carried out *in vivo* experiments based on syngeneic mouse models of colon cancer in BALB/c mice and chemically colon tumorigenesis. Flow cytometry, RNAseq, RT-qPCR, antibody-specific immune cell depletion, and Western blot were used to identify the immune cell type involved in the preventive and antitumor activity of H89. We demonstrated that H89 delays colon oncogenesis and prevents tumor growth. This latter effect seems to involve NK cells. H89 also inhibits colon tumor growth in a T-cell-dependent manner. Analysis of the immune landscape in the tumor microenvironment showed an increase of CD4^+^ Th1 cells and CD8^+^ cytotoxic T cells but a decrease of CD4^+^ T_reg_ cell infiltration. Mechanistically, we showed that H89 could promote naïve CD4^+^ T-cell differentiation into Th1, a decrease in T_reg_ differentiation, and an increase in CD8^+^ T-cell activation and cytotoxicity *ex vivo*. Furthermore, H89 induced overexpression of genes involved in antitumor immune response, such as IL-15RA, which depletion counteracts the antitumor effect of H89. We also found that H89 regulated Akt/PP2A pathway axis, involved in TCR and IL-15 signaling transduction. Our findings identify the H89 as a potential strategy for immune system activation leading to the prevention and treatment of CRC.

## Introduction

Colorectal cancer (CRC) is the third most common cancer worldwide with an incidence rate of 10.2% and a mortality rate of 9.2% among the 10 most common types of cancer (1–3). Conventional treatments for CRC essentially rely on surgery, chemotherapy, and radiotherapy used alone or in combination (4–7). The main chemotherapies used in CRC are 5-fluorouracil (5-FU) associated with oxaliplatin (8). Recently, immunotherapy emerged as an alternative way to fight cancer. This approach consists of the reactivation of the immune system exhausted by tumor cells and the tumor microenvironment (TME). Attention was first focused on CD8^+^ T lymphocytes (TLs), endowed with direct cytotoxic activity on cancer cells orchestrated *via* granzyme B (GMB)/perforin or Fas/FasL (9). Several studies have shown a positive correlation between CD8^+^ TL infiltration within tumors and the survival of patients with CRC (10, 11). Moreover, the presence of CD4^+^ TLs is also essential for the establishment of an effective response (12). The cytokine environment is fundamental for the polarization of naïve CD4^+^ TLs into different subpopulations exhibiting anti- or protumoral effects, including type 1 T-helper (Th1), Th2, and regulatory T (T_reg_) (13, 14). Cell-based approaches, mainly immune checkpoint (ICP) blockade, represent an important tool of immunotherapy. ICP are receptors involved in the modulation of the activation of immune cells to limit the duration and the intensity of immune responses (15). The cytotoxic T-lymphocyte antigen 4 (CTLA-4) and the programmed death 1 (PD-1) are frequently expressed on tumor cells to overcome immunosurveillance by suppressing T-cell function and proliferation (16). ICP blockers targeting PD-1 and its ligand PD-L1 have been successfully implemented in the therapeutic management of multiple types of cancer, giving substantial benefits to patients (17, 18). However, a limited number of patients respond to these immunotherapies, including CRC patients. To circumvent these issues, we have focused our attention on other agents that could exhibit an immunomodulatory potential such as the protein kinase (KI) *N*-[2-[[3-(4-bromophenyl)-2-propenyl]amino]ethyl]-5-isoquinoline sulfonamide (H89). This drug is known to inhibit protein kinase A (PKA) and some other kinases, including MSK1, S6K1, and ROCK-II (19, 20). Our previous reports showed that H89 could trigger human colon cancer cell death *in vitro* when combined with the nitric oxide donor glyceryl trinitrate (21, 22).

Here, we report that the KI H89 delays the development of endogenous colon carcinogenesis and reduces the growth of other transplantable mouse models of colon cancer, which rely on the activation of NK and T-cell antitumor response, respectively. These findings identify the KI H89 as a potential strategy for the prevention and treatment of colon cancer.

## Materials and Methods

### Cell Lines and Treatments

Mouse colorectal cancer cells (CT26) or triple-negative breast cancer cells (4T1) and human leukemic cells (Jurkat, MOLT-4) were purchased from the American Type Culture Collection (ATCC, Manassas, VA, USA). Mouse colorectal cancer cells (C51) were from MP Colombo (Istituto Nazionale Tumori, Milan, Italy). All cell lines were periodically tested to avoid mycoplasma contamination using a universal mycoplasma detection kit (ATCC). Cells were cultured in RPMI (CT26, C51, Jurkat, MOLT-4) or DMEM high glucose (4T1) media supplemented with 10% of fetal bovine serum (FBS) (Dominique Dutscher, Bernolsheim, France) at 37°C with 5% of CO_2_. Cells were treated with 10 µM of H89 (Cayman Chemicals, Tallinn, Estonia) at different time points.

### Tumor Models and Treatments

Seven-week-old female BALB/c mice or *Swiss nude* mice were purchased from Charles River Laboratory (Saint-Germain-Nuelles, France) or the animal care facility at the University of Burgundy (Dijon, France). Mice were inoculated with subcutaneous injection in the right flank, and under isoflurane anesthesia, with 5 × 10^5^ colon CT26, C51, or 4T1 cell lines. After 6 days, when tumors reached ≈50 mm^3^, animals were randomly assigned to different groups (5 or 7 mice per cage) and treated with H89 (10 mg/kg in 100 µl of NaCl) with intraperitoneal (i.p.) injection twice a week. For TL CD8^+^ depletion, an anti-CD8a antibody (BioXCell, Euromedex, Mundolsheim, France) 500 µg in 100 µl NaCl or IgG2A control isotype (BioXCell) 500 µg in 100 µl of NaCl was injected (i.p.) together with the first H89 administration and renewed once a week. For IL-15RA blockade, an anti-TMB1 (BioXCell (catalog number BE0298) 50 µg in 100 µl of NaCl) or IgG2A control isotype (BioXCell 50 µg in 100 µl of NaCl) was injected (i.p.) the day before the first H89 administration and renewed twice a week. For the H89/5-FU combination, 5-FU (5 mg/kg in 100 µl NaCl) was injected (i.p.) weekly at the same time as H89 administration. For the oral administration, H89 was given twice a week by gavage (5 mg/kg in NaCl). For prophylaxis experience, H89 was administrated by i.p. injections (10 mg/kg in NaCl) 3 days before CT26 implantation. Tumor growth was monitored three times a week. For AOM/DSS-induced carcinogenesis, colorectal polyps were induced by azoxymethane (AOM) and dimethylhydrazine (DMH) i.p. injections before dextran sulfate sodium (DSS) administration in drinking water according to the protocol by Rosenberg et al. (23). For this experience, mice were treated with H89 (5 mg/kg in 100 µl NaCl by gavage) twice a week. Animals’ health status was monitored by observing weight variations three times a week, and mice were killed if their weight loss was higher than 20% of their initial weight. After 90 days, mice were killed and colorectal polyps were quantified.

### NK Cell Depletion *In Vivo*

NK cell depletion was performed using an anti-Asialo GM1 antibody (FUJIFILM Wako Chemicals, Neuss, Germany). BALB/c mice received the anti-Asialo GM1 antibody (10 µl/100 µl NaCl by i.v. injection according to the supplier’s instructions) on days −5, −3, and −1 before and then every 5 days after CT26 cell injection. H89 was injected three times from D-3 to D-1. Tumor growth was monitored three times a week.

### Animal and Ethics Statement

The animal care staff observed the animals’ state each day, and we monitored their behavior. When the volume of the tumor reached 2,000 mm^3^, animals were killed. The experiments were approved by the ethics committee “C2EA Grand Campus Dijon No. 105” and by the ministry of research with the protocol codes 28546 and 33240.

### Characterization of CD4^+^ and CD8^+^ T-Cell Infiltration in the TME

CT26 tumor-bearing mice were treated with H89 (10 mg/kg in 100 µl of NaCl, i.p.) for 10, 14, or 21 days (10 mice/time point). At each time point, mice were killed and tumors were dissociated with collagenase and DNase solution before lysis of red blood cells. For membrane staining, cells were incubated with Flow Cytometry Staining Buffer (FCSB) for 15 min and then for 30 min at 4°C with membrane antibodies. For intracellular staining, cells were fixed and permeabilized with the Cytofix/Cytoperm™ kit from BD Biosciences (Le Pont de Claix, France) before incubation with antibodies for 30 min at 4°C. One million of cells were stained with viability FVS 700 (BD Biosciences) and TL-specific antibody panels (**Supplementary Tables S2****–****S4**) and analyzed by flow cytometry. All the acquisition were performed using a BD FACS Canto or an LSRII cytometer using the BD FACSDiva software (BD Biosciences). Data were analyzed using the FlowJo software v10.

### RNA Sequencing Analysis

Total RNA extraction was performed as per the manufacturer’s instructions. Sequencing, data quality, and reds repartition were performed by the BGIseq500 platform (BGI-Shenzhen, China). Differential expression analysis was performed with the DESeq2 R package (https://doi.org/10.1186/s13059-014-0550-8). Treatment groups were compared. Raw *p*-values associated with each gene were adjusted using Bonferroni correction, as advised. Gene Set Enrichment Analysis (GSEA) was used to identify biological pathways that are enriched in the lists of differential gene characteristics of each group of patients. Pathways from the Hallmark database were used. GSEA was conducted using the clusterProfiler R package (https://doi.org/10.1089/omi.2011.0118).

### Isolation of Primary Mouse Immune Cells

Naïve CD4^+^ or CD8^+^ T cells were isolated from the spleen and lymph nodes of female Balb/c mice using Miltenyi isolation kits (Miltenyi Biotech, Paris, France). Naive CD4^+^ T cells (CD62L^+^) were differentiated into Th1 or T_reg_ cell subtypes using cytokines and blocking antibody cocktails: anti-IL-4 (10 µg/ml) and IL-2 (10 µg/ml) for Th1 and anti-IFN-γ (10 µg/ml), anti-IL-4 (10 µg/ml), and TGFβ (4 ng/ml) for T_reg_ cells (24). During T-cell differentiation, Th1 and T_reg_ cells were treated with 1 µM of H89 for 72 h and T-cell differentiation was assessed by RT-qPCR. Total CD4^+^ T cells (CD62L^−^) were obtained using the negative fraction and were treated with 1 µM of H89 for 2 h to assess intracellular potassium [K^+^]_i_ concentration, as previously reported (25). CD8^+^ T cells were cultured for 72 h with 1 µM of H89 to assess the level of Akt phosphorylation and granzyme B production. All T cells were activated using anti-CD3/anti-CD28 Dynabeads (Gibco - Thermo Fischer Scientific, Waltham, MA, USA) and cultured in antibiotics-enriched RPMI medium [10% FBS with 1% penicillin, streptomycin, and amphotericin B (PSA)].

### CD8^+^ T-Cell Cytotoxic Assay

B16-OVA melanoma cancer cells were seeded in a 96-well plate (30,000 cells per well) in 200 µl of RPMI 10%FBS 1%PSA medium after cell trace™ violet labeling (Invitrogen - Thermo Fischer Scientific). Splenic CD8^+^ T cells were isolated from OT-I female mice using the CD8a^+^ T-cell Isolation Kit and cocultured with B16-OVA cells at different ratios of B16-OVA : CD8^+^ T cells) and treated, or not, with 5 µM of H89. After 48 h of coculture, B16-OVA cell viability was analyzed by flow cytometry using a Fixable Viability stain 700 (FVS 700) (BD Biosciences) and using the cell trace™ violet labeling to differentiate B16-OVA cells from CD8^+^ T cells.

### PP2A Enzymatic Activity Assay

Jurkat and MOLT-4 cells were lysed with a buffer containing imidazole-HCl, EDTA, and EGTA (Sigma-Aldrich, Saint-Quentin-Fallavier, France) with a protease and phosphatase inhibitor cocktail (Roche Life Science, Penzberg, Germany). After protein quantification with the Bio-Rad DC protein assay as recommended by the manufacturer (Bio-Rad, Marnes-la-Coquette, France), the enzymatic activity of the phosphatase PP2A was assessed using the PP2A Immunoprecipitation Phosphatase Assay Kit (Millipore, Guyancourt, France) as per the manufacturer’s procedures.

### RT-qPCR

Th1 and Treg cells as well as mice tumors were collected using the Trizol reagent (Ambion - Thermo Fischer Scientific) to perform total RNA extraction. Five hundred nanograms of total RNA were reverse transcribed into cDNA using a M-MLV reverse transcriptase, recombinant RNasin^®^ Plus RNase inhibitor, and random primers (Promega, Charbonnières-les-Bains, France). cDNA level was assessed by real-time PCR using PowerUpTM SYBR™ Green (Applied biosystems - Thermo Fischer Scientific) on ViiA™7 Real-Time PCR System (Applied biosystems). Relative mRNA expression was quantified using the 2^−ΔCt^ formula after actin normalization (ΔCt). Primers were purchased at (Eurogentec, Seraing, Belgium) and are listed in **Supplementary Table S1**.

### Immune Checkpoint Analysis

For PD-1, PD-L1, and CD80 expression analysis, CT26 or MOLT-4 cells, treated or not *in vitro* with H89, were stained using the tumor membrane staining protocol described above. All the antibodies used in this paper are listed in **Supplementary Table S5**. All the acquisitions were performed using a BD FACS Canto or an LSRII cytometer using the BD FACSDiva software (BD Biosciences). Data were analyzed using the FlowJo software v10.

### Immunohistochemistry

CT26 tumors collected from BALB/c mice were cut in 4-µm-thick slices after formalin fixation and paraffin embedding. After demasking in a water bath with Tris-EDTA buffer (20 min, 95°C, pH 9), the slices were left to cool for 10 min at RT. Inhibition of endogenous horseradish peroxidase (HRP) was performed with H_2_O_2_ (3%) in PBS followed by saturation in TBS-Tween BSA 3%. Slices were then incubated for 1 h at RT with an anti-CD4 (dilution 1:800) or anti-CD8 antibody (dilution 1:500) (HistoSure HS-360108 or 361008) and then with a secondary antibody (Impress anti-rabbit IgG 5+L, MP-7401-50) ( Vector® Laboratories, Burlingame, CA, USA) with a substrate kit HRP (Vector^®^ Laboratories, NovaRED Substrate Kit SK-4800). The samples were then exposed to a Harris hematoxylin bath for 10 s before dehydration, followed by mounting in an organic medium.

### Western Blot

Cell lysates were prepared and quantified, and proteins were separated in a 10% SDS-PAGE gel and transferred on a nitrocellulose membrane, as previously described (26). Membranes were incubated overnight at 4°C with anti-pAkt or anti-Akt antibodies (Cell signaling, Danvers, MA, USA) and HSC70 as an endogenous loading control (Santa Cruz Biotechnology, Dallas, TX, USA). Membranes were washed and incubated for 1 h with HRP-conjugated secondary antibody (Jackson Immunoresearch Laboratories, West Grove, PA, USA) and revealed using the Clarity™ Western ECL Substrate (Bio-Rad) and the ChemiDoc imaging system (Bio-Rad).

### Statistics

Statistical analyses were performed using paired or unpaired Student’s *t*-test one or two-tailed with significance determined at *p* ≤ 0.05 for *in vitro* and *ex vivo* experiments. For *in vivo* experiments, we used two-way ANOVA with Bonferroni’s correction for multiple comparisons. The GraphPad Prism 7 software was used to all statistical analyses, and differences were considered statistically significant at ^*^p ≤ 0.05, ^**^p ≤ 0.01; ^***^p ≤ 0.001; ^****^p ≤ 0.0001.

## Results

### H89 Delays AOM/DSS-Driven Carcinogenesis and Prevents Tumor Growth

**Figure 1:**
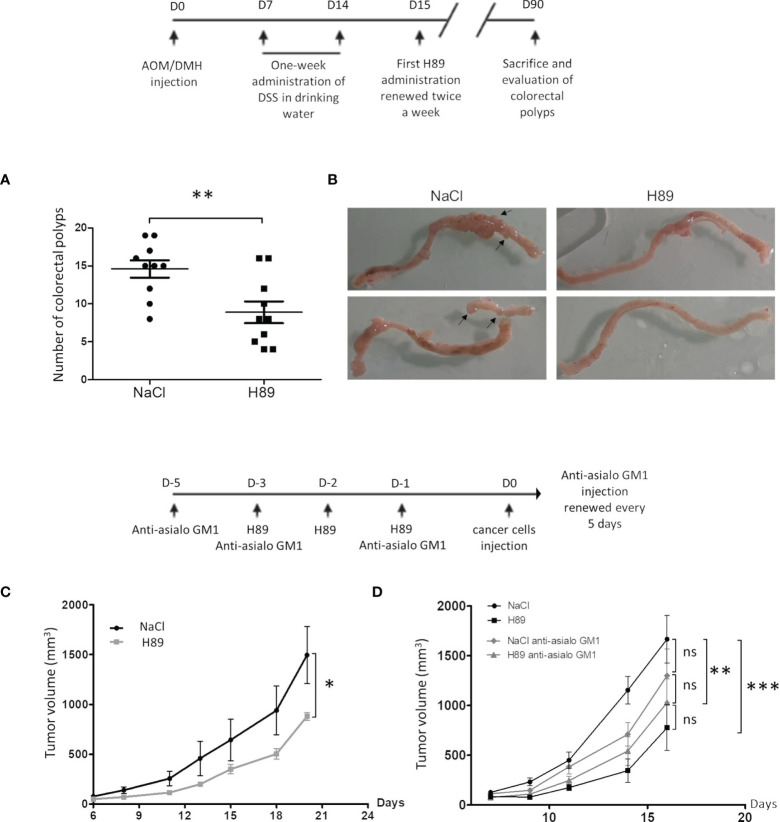
H89 delays tumor growth and AOM/DSS-driven carcinogenesis. **(A)** Macroscopic evaluation of the number of colorectal polyps in BALB/c mice with AOM/DSS-induced colorectal carcinoma, treated or not with H89 (5 mg/kg, oral administration) twice a week (*n* = 10 mice/group). **(B)** Macroscopic images of mouse colons treated or not with H89. **(C)** To assess the preventive effect of H89 in CT26-bearing BALB/c mice, H89 (or NaCl in the control group) was injected for 3 days in a row (10 mg/kg, i.p.). On day 4, 5 × 10^5^ CT26 cells were injected in s.c., and tumor growth was monitored three times a week (*n* = 5). **(D)** Two days before the first H89 injection, mice were treated with an anti-asialoGM1 to deplete NK cells (10 µl in 100 µl of NaCl according to the supplier’s instructions). Anti-Asialo GM1 Injections were repeated during the first and the last H89 injections, and then every 5 days (*n* = 7 mice/group). Statistically significant differences were determined by using a *t*-test **(A)** or two-way ANOVA **(C, D)**; ^*^p ≤ 0.05; ^**^p ≤ 0.01; ^***^p ≤ 0.001.

Based on our previous results, which showed that H89 reduces intestinal inflammation in a murine model of DSS-induced colitis (27), we investigated its prophylactic potential. In a mouse model of AOM/DSS colitis-associated intestinal carcinogenesis, we showed that H89 decreases tumor incidence as attested by a significant reduction in the total number of colorectal polyps (**Figures 1A, B**). In parallel, using a CT26 tumor-bearing mouse model, mice receiving H89 for 3 days in a row prior to implantation of CT26 cells, showed a significant decrease in tumor incidence (**Figure 1C**). We hypothesized that such an effect might involve an early innate immune response as NK cells. As shown in **Figure 1D**, we observed a significant antitumor effect in mice receiving anti-Asialo GM1 (used to deplete NK cells) compared with the control. Such effect is less pronounced than in H89-treated mice. However, the combination of H89 with anti-Asialo GM1 tends to inhibit H89-mediated tumor growth prevention but not to a significant extent. This result suggested that the preventive effect of H89 may involve other innate immune cells since the combination also significantly prevented tumor growth (**Figure 1D**).

### H89 Mediates Immunotherapeutic Activity Against Colon Cancer

**Figure 2:**
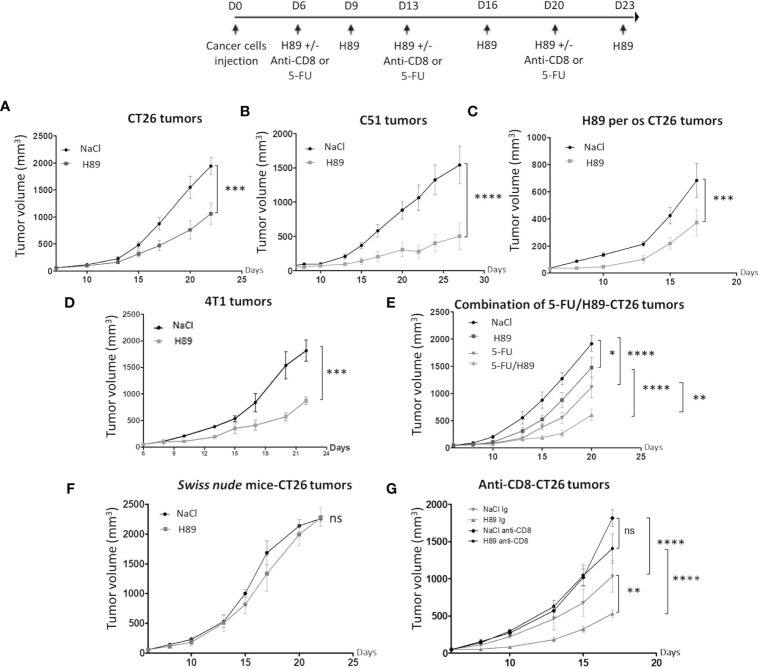
H89 mediates immunotherapeutic activity against colon cancer. **(A, B)** CT26 or C51 tumor-bearing BALB/c mice (5 × 10^5^ murine colon cancer cells in s.c.) were treated, or not (NaCl, i.p.), with H89 (10 mg/kg, i.p.) twice a week, and tumor growth was monitored three times a week (**A**, *n* = 10 mice/group; **B**, *n* = 7 mice/group). **(C)** Oral administration of H89 (5 mg/kg), or NaCl in the control group, twice a week in CT26 tumor-bearing BALB/c mice. Tumor growth was monitored three times a week (*n* = 7 mice/group). **(D)** 4T1 breast cancer cells tumor-bearing mice (5 × 10^5^ 4T1 cells in s.c.) were treated, or not (NaCl, i.p.), with H89 (5 mg/kg, i.p.) twice a week, and tumor growth was monitored three times a week (*n* = 5 mice/group). **(E)** CT26 tumor-bearing BALB/c mice (5 × 10^5^ CT26 cells in s.c.) were treated, or not (NaCl, i.p.), with H89 (10 mg/kg, i.p.) twice a week, in combination with 5-fluorouracil (5-FU, 5 mg/kg in i.p.) once a week, and tumor growth was monitored three times a week (*n* = 14 mice/group). **(F)** Swiss nude immunodeficient mice, bearing CT26 tumors (5 × 10^5^ CT26 cells in s.c.) were treated, or not (NaCl, i.p.), with H89 (10 mg/kg, i.p.) twice a week, and tumor growth was monitored three times a week (*n* = 5 mice/group). **(G)** CT26 tumor-bearing BALB/c mice (5 × 10^5^ CT26 cells in s.c.) were treated, or not (NaCl, i.p.), with H89 (10 mg/kg, i.p.) twice a week. Mice also received anti-CD8a or control IgG injections once a week (500 µg in i.p.), and tumor growth was monitored three times a week (*n* = 7 mice/group). Statistically significant differences were determined by using two-way ANOVA: ^*^p ≤ 0.05; ^**^p ≤ 0.01; ^***^p ≤ 0.001; ^****^p ≤ 0.0001; n.s., nonsignificant results.

Besides its prophylactic potential, we investigated whether H89 may have an antitumor therapeutic effect. We investigated the role of the adaptive immune system on mouse models of colon cancer. Intraperitoneal or per os administration (one route of administration used in anticancer treatments in humans) of H89 in BALB/c mice bearing CT26 or C51 cells significantly decelerated tumor progression (**Figures 2A–C**). Such antitumor effect of H89 was not restricted to colon cancer and was also observed in a triple-negative breast cancer mouse model, known for its aggressiveness and resistance to treatment (**Figure 2D**). We also investigated whether H89 may potentiates chemotherapy used in CRC such as 5-FU. Importantly, even though each agent used alone reduced CT26 tumor growth, their combination significantly enhanced their antitumor capacity (**Figure 2E**). We further tested whether H89-mediated tumor growth inhibition depends on the immune system. We then induced tumors in *Swiss nude* mice, lacking T cells, and treated them with H89. The monitoring of tumor growth showed that the antitumoral activity of H89 is stopped in *Swiss nude* mice (**Figure 2F**). We also showed that the depletion of CD8^+^ TLs blunted the antitumor efficacy of H89 (**Figure 2G**). We also observed that the anti-CD8^+^ antibody alone increased tumor growth compared with the vehicle, indicating the suppressive function of CD8^+^ TLs on tumor growth. Altogether, our results reveal that H89 antitumor efficacy is dependent on an adaptive immune response.

### H89 Increases CD8^+^ TL Tumor Infiltration, Activation, and Function

**Figure 3:**
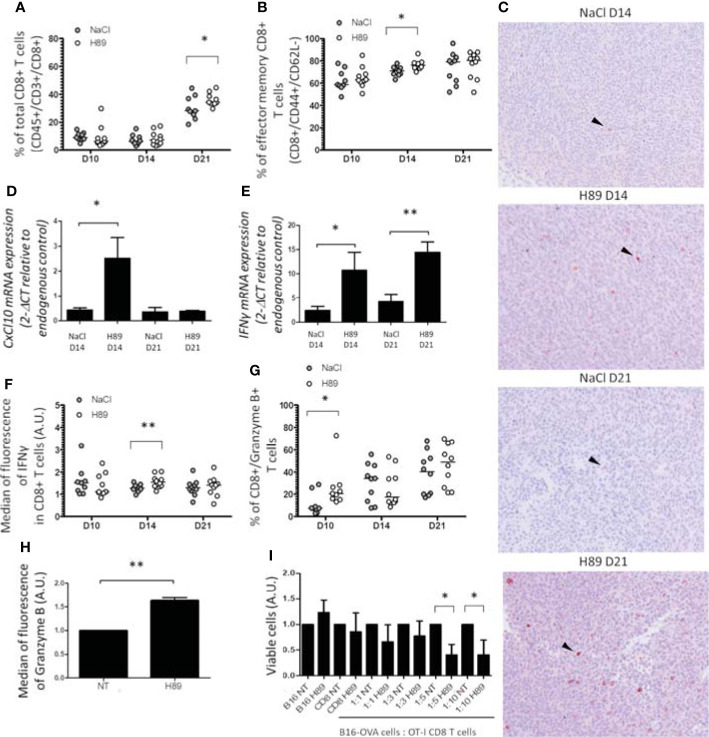
H89 increases CD8^+^ TL tumor-infiltration, activation, and function. **(A, B)** Flow cytometry analysis (*n* = 10 mice/group) of the intratumor infiltration of CD8^+^ T cells under H89 treatment on day 10 (D10), D14, and D21 after CT26 colon cancer cells injection (5 × 10^5^ in s.c.). BALB/c mice were treated or not (NaCl) by H89 (10 mg/kg, i.p. administration twice a week). **(C)** IHC analysis on CT26 tumors after CD8^+^ T-cell labelling at D14 and D21 (representative images of 3 animals/group/time point). RT-qPCR analysis of *CXCL10* **(D)** and *IFN-γ* **(E)** expression at D14 and D21 after CT26 cancer cell injection (*n* = 3 mice) and H89 treatment (10 mg/kg, i.p., NaCl in the control group). The results are presented as the mean of 2^−ΔCT^ values. **(F, G)** Flow cytometry analysis after intracellular labeling (*n* = 10 mice/group) of IFN-γ and granzyme B in intratumor CD8^+^ T cells at D10, D14, and D21 after CT26 colon cancer cell injection and H89 treatment (10 mg/kg, i.p., NaCl in the control group). **(H)** Flow cytometry analysis of granzyme B production of splenic CD8^+^ T cells *ex vivo* after H89 treatment (5 µM, 72 h) (*n* = 3) and **(I)** cytotoxic assay using CD8^+^ T cells from OT-I mice cocultured with B16-OVA cells *in vitro* treated by H89 (5 µM, 48 h) (*n* = 3). **(H–I)** The results are calculated as the mean of 2^−ΔCT^ values. Each control values (NT) are set at 1 arbitrary unit (A.U.), and H89-treated conditions are compared with the control. Statistically significant differences were determined by using a *t*-test: ^*^p ≤ 0.05; ^**^p ≤ 0.01.

We investigated the effect of H89 on the intratumoral infiltration of CD8^+^ TLs and their state of activation and function. Quantification of intratumoral CD8^+^ TLs by flow cytometry analysis revealed that H89 significantly increased the amount of total CD8^+^ TLs (CD45^+^/CD3^+^/CD8^+^ cells) on day 21 but not on days 10 and 14 posttumor cell injection (**Figure 3A**). We found that H89 significantly increases the number of effector memory CD8^+^ TLs on day 14 but not the central memory TLs (**Figure 3B**; **Supplementary Figure S1**). Such effects were confirmed by IHC staining (**Figure 3C**). To depict how H89 mediates tumor recruitment of CD8^+^ TLs, we analyzed by RT-PCR the mRNA expression level of the chemokine *CXCL10*, known to attract CD8^+^ TLs in the TME. The results showed that the H89 significantly increased the expression of the *CXCL10* gene transcript on day 14 (**Figure 3D**). We also analyzed the impact of H89 on the activation of CD8^+^ TLs by measuring interferon-gamma (IFN-γ) and granzyme B expression. We showed that H89 significantly increases the amount of IFN-γ mRNA within the tumor and IFN-γ production in CD8^+^ TLs (**Figures 3E, F**). Regarding granzyme B, H89 significantly raised its expression on day 10 (**Figure 3G**). A similar effect was obtained when CD8^+^ TLs isolated from the spleen lymph nodes of BALB/c mice were treated *in vitro* with H89 for 72 h (**Figure 3H**). We next used the OT-I/B16-OVA mouse model to investigate whether the activation of CD8^+^ by H89 correlates with their cytotoxic activity. Thus, we investigated the administration of H89 on *ex vivo* cocultured CD8^+^ TLs, purified from OT-I mice, with melanoma B16-OVA for 48 h significantly decreased melanoma B16-OVA cell viability in 1:5 and 1:10 ratios (target:effector) (**Figure 3I**).

### Effect of H89 on CD4^+^ TL Infiltration and Differentiation

**Figure 4:**
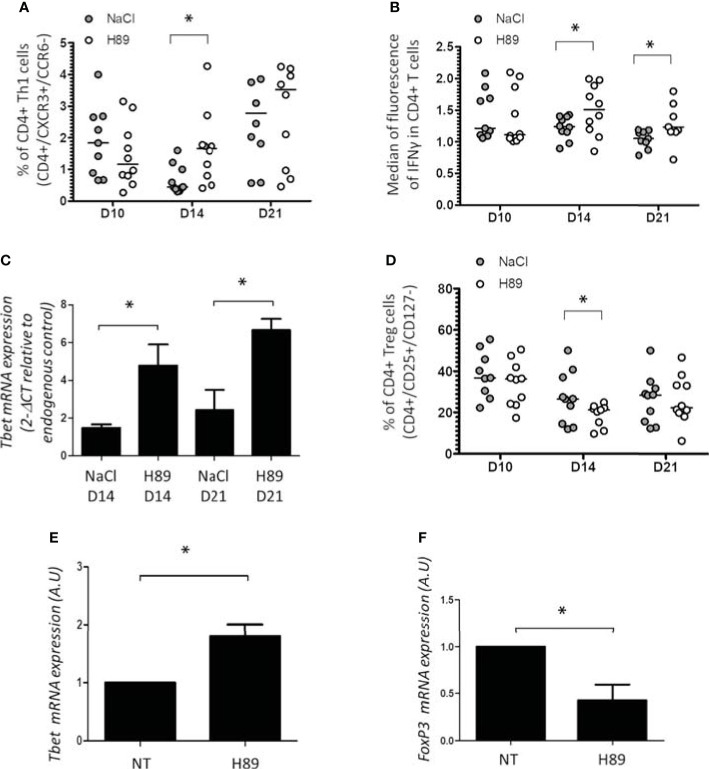
Effect of H89 on CD4^+^ TL infiltration and differentiation.** (A, B, D)** Flow cytometry analysis (*n* =10 mice/group) of the intratumor infiltration of CD4^+^ Th1, Treg, and IFN-γ expression in CD4^+^ T cells on day 10 (D10), D14, and D21 after CT26 colon cancer cell injection into BALB/c mice (5 × 10^5^ in s.c.), treated or not by H89 (10 mg/kg, i.p. injection two times a week). Control group received NaCl injection. **(C)** RT-qPCR analyses of *Tbet* expression at D14 and D21 after CT26 cancer cell injection into BALB/c mice (*n* =3 mice). The results are presented as the mean of 2^−ΔCT^ values. **(E, F)** RT-qPCR analyses of *Tbet* and *FoxP3* expression on splenic naive CD4^+^ T cells from BALB mice after *in vitro* differentiation into Th1 and Treg under H89 treatment (1 µM, 72 h) (*n* = 3). The results are calculated as the mean of 2^−ΔCT^ values. Each control values (NT) are set at 1 arbitrary unit (A.U.) and H89-treated conditions are compared with control. Statistically significant differences were determined by using a *t*-test: ^*^p ≤ 0.05.

In parallel, we investigated CD4^+^ TL infiltrates in H89-treated and H89-untreated mice bearing CT26 tumors. We showed that H89 significantly increases the amount of intratumoral CD4^+^ TLs (CXCR3^+^/CCR6^−^) (Th1) expressing IFN-γ (CD4^+^/IFN-γ^+^ TLs) (**Figures 4A, B**). In addition, we confirmed the intratumoral presence of Th1 cells by RT-qPCR analysis of *T-bet* expression, a well-known key transcription factor of Th1 cell differentiation. Indeed, H89 significantly increased the expression of *T-bet* on days 14 and 21 (**Figure 4C**). Conversely, CT26 tumors treated with H89 exhibited a decreased level of CD4^+^ T_regs_ (CD25^+^/CD127^−^) (**Figure 4D**). However, we failed to detect significant differences in Th2 (IL-4^+^ TLs) and Th17 (IL-17^+^ TLs) from untreated versus H89-treated tumors (**Supplementary Figure S2A, B**). We also evaluated the ability of H89 to induce the differentiation of Th1 and Treg cells *in vitro*. Differentiation of CD4^+^ naïve T cells isolated from the spleen and lymph nodes of BALB/c mice showed that H89 promotes Th1 differentiation as attested by a significant increase of *T-bet* transcription (**Figure 4E**). Conversely, H89 did not promote T_reg_ cell differentiation attested by the inhibition of *FoxP3* gene expression (**Figure 4F**).

### H89 Modulates Immunosuppressive Receptors Expressed Either by T Cells or Colon Cancer Cells

**Figure 5:**
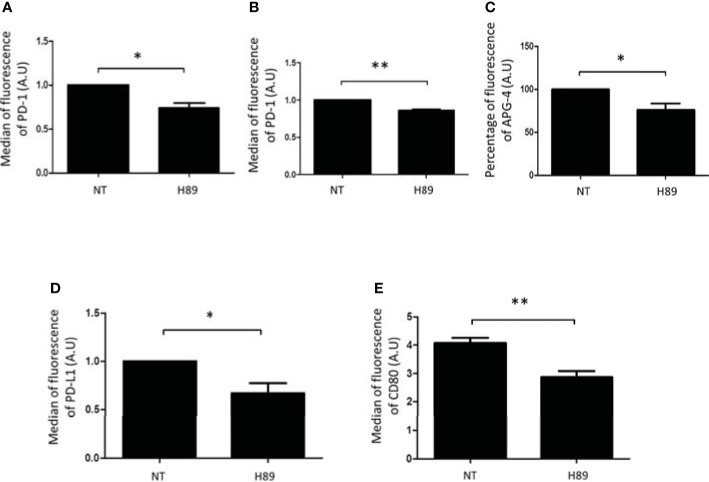
H89 modulates immunosuppressive receptors expressed either by T cells or colon cancer cells. Flow cytometry analysis of PD-1 expression on CD4^+^ T cells isolated from the spleen of BALB/c mice **(A)** or MOLT-4 cells **(B)** cells treated by H89 for 24 h (*n* = 3). Flow cytometry intracellular K^+^ detection using APG-4 probe on splenic CD4^+^ T cells treated by H89 for 2 h (*n* = 3) **(C)**. Flow cytometry analysis of PD-L1/CD80 expression on CT26 colon cancer cells treated by H89 for 24 h (*n* = 3) **(D, E)**. Statistically significant differences were determined by using a *t*-test: ^*^p ≤ 0.05; ^**^p ≤ 0.01.

We analyzed by flow cytometry the impact of H89 on some immunosuppressive molecules expressed at the surface of TLs or cancer cells. We found that H89 significantly decreased the expression of PD-1 on total CD4^+^ TLs (isolated from the spleen of BALB/c mice) and on the CD8^+^-like cell line MOLT-4 (**Figures 5A, B**). We also analyzed another ICP, the intracellular K^+^ as a marker of TL activation. Indeed, naive or exhausted TLs are characterized by a high intracellular level of K^+^; this level decreases when TLs are activated (25). We then evaluated the impact of H89 in [K^+^]_i_ regulation in CD4^+^ cells. We observed that CD4^+^ TLs exhibited a low level of [K^+^]_i_ in presence of H89 (**Figure 5C**), attesting to their activation. Since some receptors such as PD-L1, another immunosuppressive actor, and CD80 (exhibiting activating and immunosuppressive functions according to the conditions) can be expressed on the cellular surface of cancer cells, we have tried to find out the influence of H89 on the expression of these receptors on the surface of CT26 cells. As depicted in **Figure 5**, H89 significantly affected both PD-L1 (**Figure 5D**) and CD80 (**Figure 5E**) expression.

### H89 Regulates Signaling Pathways Involved in Immune Cell Activation and Cancer Cell Growth

**Figure 6:**
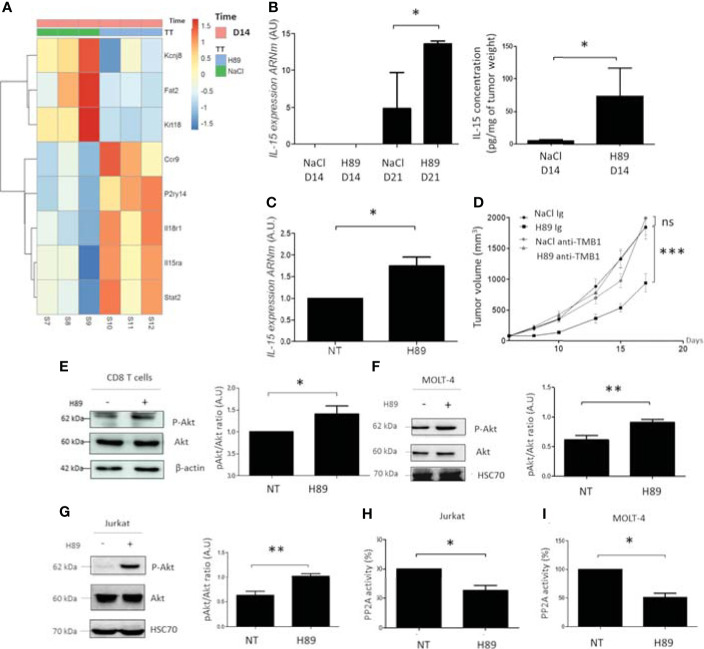
H89 regulates signaling pathways involved in immune cell activation and cancer cell growth. **(A)** RNAseq analysis were performed on CT26 solid tumors isolated from BALB/c mice treated or not (NaCl) with H89 (10 mg/kg, i.p.) at D14 after colon cancer cells injection (*n* = 3 mice/group). **(B)** RT-qPCR analysis of *IL-15* expression mRNA level at D14 and D21 after CT26 cancer cell injection into BALB/c mice (*n* = 3 mice/group) and ELISA analysis of IL-15 on tumor lysates ad D14 (*n* = 5 mice/group). **(C)** RT-qPCR analysis of *IL-15* expression mRNA level on CT26 cells treated *in vitro* with H89 for 24 h (*n* = 3). **(D)** CT26 tumor-bearing BALB/c mice (5 × 10^5^ CT26 cells in s.c.) were treated, or not (NaCl, i.p.), with H89 (10 mg/kg, i.p.) twice a week. Mice also received anti-TMB1 or control IgG injections once a week (50 µg in i.p.), and tumor growth was monitored three times a week (*n* = 7 mice/group). **(E–G)** Western blot analysis of Akt phosphorylation (P-Akt) after H89 treatment in CD8 T cells isolated from the spleen of BALB/c mice or MOLT-4 and Jurkat T cells (*n* = 5) **(H, I)** Quantification of the enzymatic activity of phosphatase PP2A in Jurkat and MOLT-4 T cells (*n* = 3). Statistically significant differences were determined by using two-way ANOVA **(D)** or a *t*-test **(B**, **C**, **E**–**I)**: ^*^p ≤ 0.05 (or 0.1 in **(B)** with 90% confidence level); ^**^p ≤ 0.01; ^***^p ≤ 0.001; n.s., nonsignificant results. RNAseq analysis was performed with the DESeq2 package using a Wald test.

To further decipher the molecular mechanisms related to the antitumor effect of H89, CT26 tumors on day 14 postinjection were harvested for transcriptomic analysis. Analysis of the transcriptome profiling of CT26 tumors from H89-treated and untreated mice led to the selection of 69 genes significantly differentially expressed based on an absolute log fold-change greater than 1 and an adjusted *p*-value below 0.05 (**Supplementary Figure S3**). Among them, 7 genes were downregulated, and 62 were overexpressed in treated mice (**Supplementary Figure S3**). Several genes significantly upregulated with H89 are related to immune function, such as IL-15RA, IL-18R1, STAT2, CCR9, and P2ry14, a G protein-coupled receptor. While some cancer-associated genes were downregulated, such as potassium channel 6.1 (Kir6.1), the atypical cadherin, FAT2, and keratin 18 (Krt18) (**Figure 6A**). Furthermore, GSEA analysis (FDR < 0.05) revealed distinct hallmark pathways between H89-treated (D14 of treatment) and untreated tumors, in particular, H89-mediated amplification of hallmarks of IFN-α and IFN-γ response (an antitumor phenotype) compared with untreated tumors with elevated protumoral hallmarks such as MYC pathway and oxidative phosphorylation (**Supplementary Figure S3B**).

Based on these transcriptomic data, we particularly focused our attention on the H89-mediated modulation of the IL-15/IL-15RA axis. Interestingly, the IL-15/IL-15RA signaling pathway is important in the activation of immune cells, including CD8^+^ TLs, and is associated with a favorable prognosis in CRC (10, 28). We first tested whether H89 also induced IL-15 in addition to IL-15RA. We found that H89 increases both mRNA and protein levels of IL-15 in tumors (**Figure 6B**). IL-15 mRNA levels were also increased *in vitro* in CT26 treated with H89 for 24 h (**Figure 6C**). Furthermore, we investigated the involvement of the IL-15/IL-15RA pathway in H89-mediated tumor growth inhibition in CT26 tumor-bearing mice. We found that blocking IL-15 signaling with an anti-TMB1 antibody abrogated the antitumor effect of H89 (**Figure 6D**). Since the Akt signaling pathway is mainly under the control of IL-15 (29), this prompted us to assess the phosphorylation status of Akt. We found that H89 significantly increases the phosphorylation of Akt (at Ser 473) in CD8^+^ TLs (**Figure 6E**), MOLT-4 cells (CD8^+^-like phenotype) (**Figure 6F**), and Jurkat cells (CD4^+^-like phenotype) (**Figure 6G**). Since Akt is a target of the serine/threonine protein phosphatase 2A (PP2A) (30), we investigated whether H89-mediated phosphorylation of Akt could be associated with the inhibition of PP2A activity. We showed that H89 significantly reduces PP2A activity in Jurkat (**Figure 6H**) and MOLT-4 cells (**Figure 6I**).

## Discussion

Here, we demonstrate that the kinase inhibitor H89 not only delays the manifestation of tumors when used as a prophylactic intervention but also mediates therapeutic effects on established murine colorectal tumors. In the first setting, one can speculate that H89 could be used as adjuvant therapy after resection of the early stage of CRC. In such a situation, only conventional chemotherapies are used as adjuvants such as 5-FU and FOLFOX (31). The introduction of immunotherapy as an adjuvant in CRC treatment is currently under study in multiple registered clinical trials (31). In this context, H89 could be a new immunotherapy strategy in its use as an adjuvant in colon cancer. Indeed, it is able to prevent tumor growth probably dependent on NK cells. To our knowledge, few molecules have this potential. Recently, it has been shown that nicotinamide, a variant of vitamin B3, currently used as a nutritional supplement delays the development of mammary carcinogenesis through NK activation (32). In our case, nothing excludes the involvement of myeloid cells such as monocytes/macrophages in this preventive effect of H89. Our results also showed that H89 exerts an antitumor effect on two models of murine CRC, with is more pronounced in one than in the other. This could be related to the microsatellite stability or instability caused by the DNA mismatch repair (MMR) system. In our case, the less-sensitive tumors are microsatellite stable (MSS), and in our hypothesis, the more sensitive could have a microsatellite instability (MSI) status, a phenotype that should be demonstrated. Concerning the therapeutic effect of H89, it depends on CD8^+^ TLs, suggesting that this kinase inhibitor is endowed with immunomodulatory activity, which is involved in its antitumor effect. Besides its antitumor effect, H89 can also potentiate the therapeutic efficacy of standard chemotherapy in the treatment of colon cancer, namely 5-FU. Our results are consistent with data showing that the use of multikinase inhibitors, e.g., regorafenib exhibits antitumor and immunomodulatory properties (33, 34). Unlike the very low number of the small molecules multikinase inhibitors tested against CRC, a myriad of other small molecules directed selected kinases involved in various pathways mediating the initiation, progression, and migration of CRC are currently being explored [for review (35)]. It is worthy to note that H89 inhibits tumor growth of CT26 cells, which is well known to share molecular features with aggressive, undifferentiated, refractory human colorectal carcinoma cells (36). During this study, we discovered that H89 acts as a therapeutic agent by the activation of the antitumor immune response. Thus, it is efficient to recruit within tumors CD8^+^ and Th1 TLs, probably by its ability to induce the chemokine CXCL10, but it alleviates the presence of T_regs_ in TME. Although we have not demonstrated whether CXCL10 is at the origin of these latter lymphocytic traffics, it is well known to be a chemokine that plays a key role in the recruitment of CD8^+^ and Th1 cells (37). Given the fact that the antitumor effect of H89 is accompanied by the recruitment of helper 1 and cytotoxic (CD8^+^) T lymphocytes, H89 could constitute a strategy to make cold colonic tumors (characterized by little immune infiltrate) hot or warm tumors (infiltrated by effector T cells). This situation could increase the antitumor response of certain nonimmunogenic chemotherapies.

In this report, we also observed that H89 not only induces the recruitment of CD8^+^ and CD4^+^ T cells at the tumor site but also allows their activation, attested by, e.g., the proinflammatory cytokine IFN-γ induction, emphasizing the establishment of immune protection against tumor development. Such immune signature (tumor infiltration and activation of CD8^+^ T cells) has been considered a powerful factor that predicts favorable prognosis in colorectal cancer patients (10, 11, 38, 39). However, it is questionable whether H89 has an anti-inflammatory function (prevention of chemically induced carcinogenesis) or a proinflammatory function (by inducing IFN-γ). In our case, H89 induced IFN-γ (a proinflammatory cytokine essentially secreted by TLs and NK cells) which can induce an antitumor effect, unlike proinflammatory cytokines released by, e.g., neutrophils or monocytes, involved in inflammatory bowel disease (IBD) (for review: 40). Our results also revealed that the involvement of an adaptive response of the antitumor effect of H89 may be related to its ability to reduce the expression of PD-L1 and CD80 on cancer cells. Results observed for CD80 lead us to ask whether CD80 plays as an inhibitor or activator co-factor. Our hypothesis that CD80 functions as an inhibitor is based on data reported by Tirapu et al. (41). The authors showed that the silencing of CD80 by RNA interference led to the loss of tumorigenicity of CT26 tumor cells in immunocompetent mice, but not in immunodeficient mice. Further, CT26 tumor cells bind CTLA-4Ig, but much more faintly with a similar CD28Ig chimeric protein, thus providing an explanation for the dominant inhibitory effects on tumor immunity displayed by CD80. In our case, reduced expression of CD80 on tumor cells by H89 may reduce the tumor-mediated exhaustion of both CD4^+^ and CD8^+^ TLs. Relief of exhaustion in TILs in CRC remains a challenge. Most CRC patients poorly respond to ICP blockers, well known to regulate the exhaustion stage of TILs. However, it has been reported that standard chemotherapy for CRC treatments such as 5-FU or oxaliplatin alone or combined modulate the level of exhaustion of TILs attested by exhibited higher effector function to reduce tumor burden (42, 43).

We have shown in this study that the combination of H89 and 5-FU improves their antitumor effect compared with the effect of each other used alone. However, we do not know whether this effect is attributed to a better antitumor immune response or to a direct influence on tumor cell growth.

Our results suggest that H89 can act directly on the tumor cell not by inducing their death as we have previously shown (22) but by making them more immunogenic. Indeed, we showed that H89 increases the mRNA and protein expression of IL-15 *in vitro* on CT26 cells and IL-15RA mRNA at the site of tumors, a signaling pathway that can promote the activation of CD8^+^ TLs and NK cells as previously reported (29, 44). We noted that there is a mismatch in the expression of mRNA versus protein in 14- and 21-day tumors. Such difference seems to be related to a transient expression of mRNA and stability in the protein expression, as reported by Harnik et al. (45). In our case, IL-15/IL-15RA was found to be involved in the antitumor activity of H89, probably by the efficacy of H89 to the expression of IL-15 and its receptor IL-15RA. This later is frequently downregulated in cancer patients, reducing the efficacy of IL-15-based treatments, hence the development of other strategies such as IL-15 superagonist receptor-linker–IL-15 (RLI), designed to bypass the need for endogenous IL-15RA, as a promising approach to stimulate host immunity (44). The advantage of the use of H89 compared to the later treatment strategies is the ability of H89 to increase the expression of both IL-15 and IL-15RA and leads to better activation of an antitumor immune response. Another evidence of a possible direct effect of H89 on cancer cells is its ability to reduce the transcripts of certain tumor markers, such as keratin 18 (Krt18) and atypical cadherin (FAT2). Of note, Krt18 is correlated with the malignant status and acts as an oncogene in colorectal cancer (46) as for FAT2, an independent prognostic factor for the poor prognosis of gastric carcinoma (47). Furthermore, H89 affects the expression of PD-L1 on cancer cells, which contributes to cancer immune evasion. Thus, by its ability to reduce the expression of these proteins, H89 can also make cancer cells more permissive to an antitumor immune response that it also activates. In summary, we discovered that the kinase inhibitor H89 is efficient at either preventing colon oncogenesis or inhibiting various mouse models of colon tumors established in immunocompetent hosts. Such effects are due to H89-mediated re-establishing of NK- and T-cell-dependent immunosurveillance, respectively, making H89 a potential strategy for the prevention and treatment of colon cancer.

## Data Availability Statement

The original contributions presented in the study are publicly available. This data can be found here: https://www.ncbi.nlm.nih.gov/geo/GSE197598.

## Ethics Statement

The animal study was reviewed and approved by C2EA Grand Campus Dijon No. 105.

## Author Contributions

SG planned and carried out the experiments, performed the analysis and interpretation, drafted the manuscript, and designed all the figures and wrote their captions. CR carried out experiments, analysis, interpretation, and revised the manuscript. NM contributed to the experiments. JS performed the RNAseq experiments. EG contributed to the experiments. EB performed the RNAseq analyses. CT performed the RNAseq analyses. LC performed the RNAseq experiments. FV contributed to the design of an *ex vivo* experiment and revised the manuscript. CP contributed to the design of an *in vivo* experiment and revised the manuscript. SP and AB designed and directed the project. The main text of the manuscript was written by AB and SP helped to edit the manuscript. All the authors revised the last version of the manuscript. All authors listed have made a substantial, direct, and intellectual contribution to the work and approved it for publication.

## Funding

This work was supported by the “La Ligue Contre le Cancer–Conférence de Coordination InterRégionale Est (CCIR Est) under grant No. 2021-0093; the Department of Genetic Medicine, Weill Cornell Medicine-Qatar (Qatar Foundation, Doha, Qatar) under grant No. NRP9-459-3-090; and the University of Burgundy and EPHE.

## Conflict of Interest

The authors declare that the research was conducted in the absence of any commercial or financial relationships that could be construed as a potential conflict of interest.

## Publisher’s Note

All claims expressed in this article are solely those of the authors and do not necessarily represent those of their affiliated organizations, or those of the publisher, the editors and the reviewers. Any product that may be evaluated in this article, or claim that may be made by its manufacturer, is not guaranteed or endorsed by the publisher.

## Supplementary Material

Supplementary Figure 1 Central memory CD8+ T cells recruitment in response to H89. Flow cytometry analysis of the intra-tumor infiltration of central memory CD8+ T cells day 10 (D10), D14 and D21 after CT26 colon cancer cells injection into BALB/c mice (5x10^5^ in s.c.), treated or not by H89 (10 mg/kg, i.p. injection two times a week). Control group received NaCl injection. (n=10 mice/group). Statistical analyses were performed using a *t-test.* *p ≤ 0,05

Supplementary Figure 2 Th2 and Th17 CD4+ T cells recruitment in response to H89. Flow cytometry analysis of the intra-tumor infiltration of Th2 **(A)** and Th17 **(B)** CD4+ T cells day 10 (D10), D14 and D21 after CT26 colon cancer cells injection into BALB/c mice (5x10^5^ in s.c.), treated or not by H89 (10 mg/kg, i.p. injection two times a week). Control group received NaCl injection. (n=10 mice/group). Statistical analyses were performed using a *t-test.*

Supplementary Figure 3 Heatmap showing differential gene expression and GSEA Hallmark pathways variation in response to H89. RNAseq analyses for gene expression **(A)** and GSEA Hallmark pathways variation **(B)** were performed on colorectal tumors from BALB/c mice treated or not with H89 at D14 after CT26 cancer cells injection (n=3 mice/group). Statistical analyses were performed using the DESeq2 package using a Wald test*. GSEA* was conducted using clusterProfiler R package.

Supplementary Figure 4 Flow cytometry dot plot representation of effector memory CD8+ T cells at D14 after H89 treatment. Representation of the gating strategy on CT26 tumors treated with H89 at D14 (10 mg/kg, i.p., NaCl in the control group) for the identification of effector memory (EM) CD8 T cells. EM CD8 T cells are highlighted in the red frame.

Supplementary Figure 5 Flow cytometry dot plot representation of CD8+/GranzymeB+ CD8 T cells at D14 after H89 treatment. Representation of the gating strategy on CT26 tumors treated with H89 at D14 (10 mg/kg, i.p., NaCl in the control group) for the identification of Granzyme B+ CD8 T cells which are highlighted in the red frame.

Supplementary Figure 6 Flow cytometry dot plot representation of Th1 and Treg cells at D14 after H89 treatment. Representation of the gating strategy on CT26 tumors treated with H89 at D14 (10 mg/kg, i.p., NaCl in the control group) for the identification of Th1 and Treg cells which are highlighted in the red frame.
