# Supplementary figures and images for: Protein Kinase Inhibitor-Mediated Immunoprophylactic and Immunotherapeutic Control of Colon Cancer
Source: Front Immunol. 2022 Apr 28;13:875764. doi: 10.3389/fimmu.2022.875764 (PMC9097540; doi:10.3389/fimmu.2022.875764)

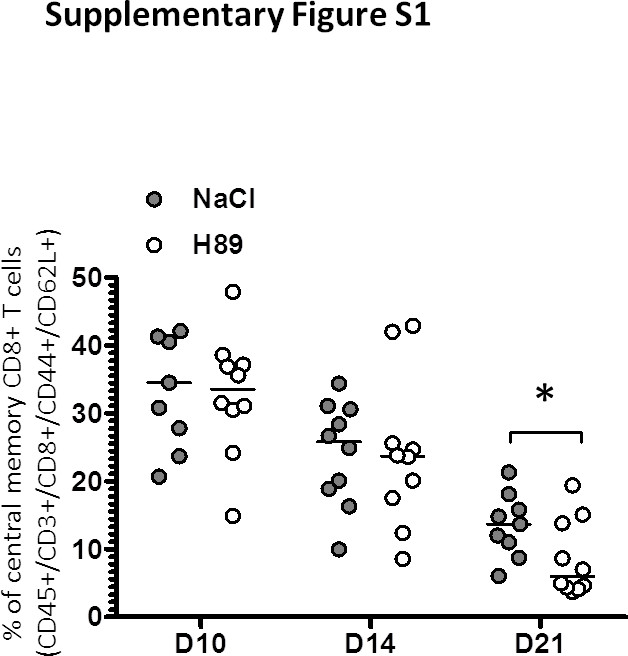

Supplement: Supplementary Figure 1 — Central memory CD8+ T cells recruitment in response to H89. Flow cytometry analysis of the intra-tumor infiltration of central memory CD8+ T cells day 10 (D10), D14 and D21 after CT26 colon cancer cells injection into BALB/c mice (5x10^5^ in s.c.), treated or not by H89 (10 mg/kg, i.p. injection two times a week). Control group received NaCl injection. (n=10 mice/group). Statistical analyses were performed using a t-test. *p ≤ 0,05 [file Image_1.jpeg]

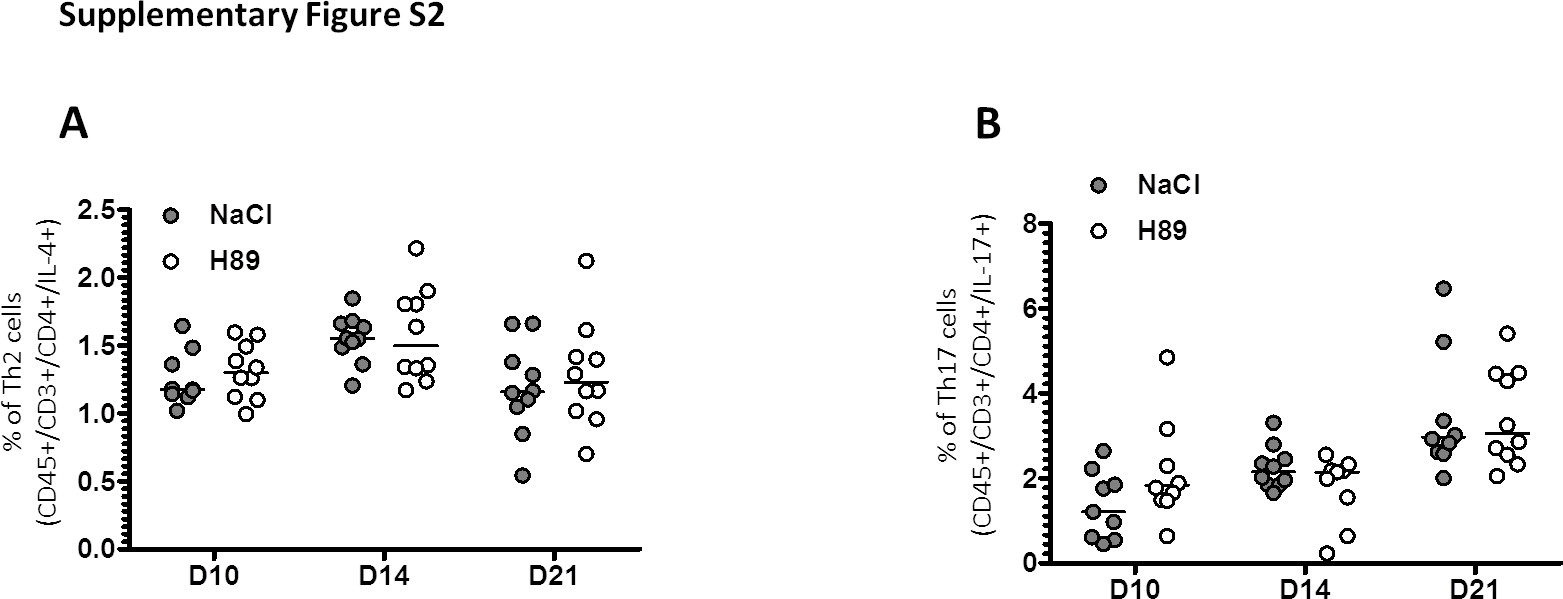

Supplement: Supplementary Figure 2 — Th2 and Th17 CD4+ T cells recruitment in response to H89. Flow cytometry analysis of the intra-tumor infiltration of Th2 (A) and Th17 (B) CD4+ T cells day 10 (D10), D14 and D21 after CT26 colon cancer cells injection into BALB/c mice (5x10^5^ in s.c.), treated or not by H89 (10 mg/kg, i.p. injection two times a week). Control group received NaCl injection. (n=10 mice/group). Statistical analyses were performed using a t-test. [file Image_2.jpeg]

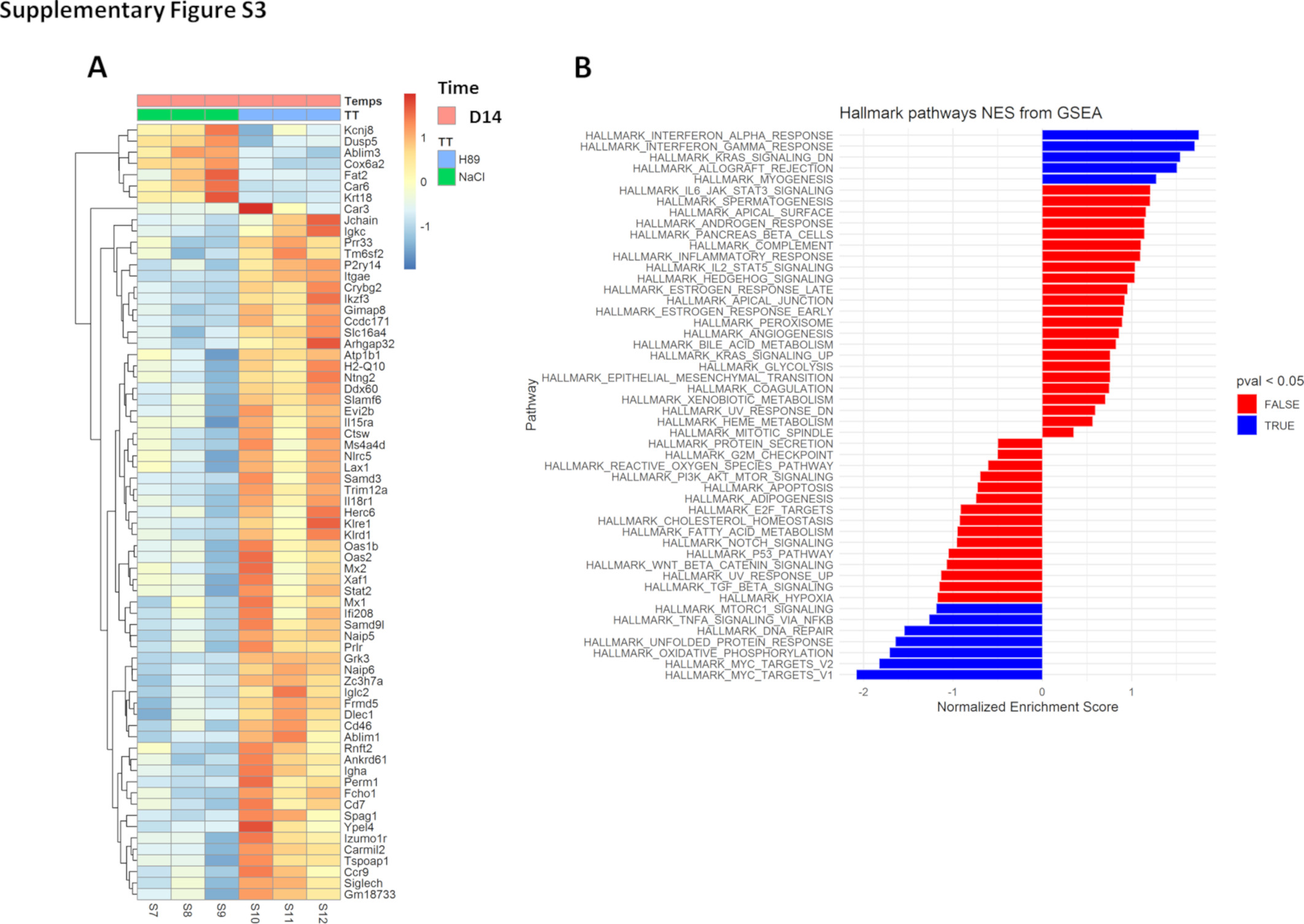

Supplement: Supplementary Figure 3 — Heatmap showing differential gene expression and GSEA Hallmark pathways variation in response to H89. RNAseq analyses for gene expression (A) and GSEA Hallmark pathways variation (B) were performed on colorectal tumors from BALB/c mice treated or not with H89 at D14 after CT26 cancer cells injection (n=3 mice/group). Statistical analyses were performed using the DESeq2 package using a Wald test. GSEA was conducted using clusterProfiler R package. [file Image_3.jpeg]

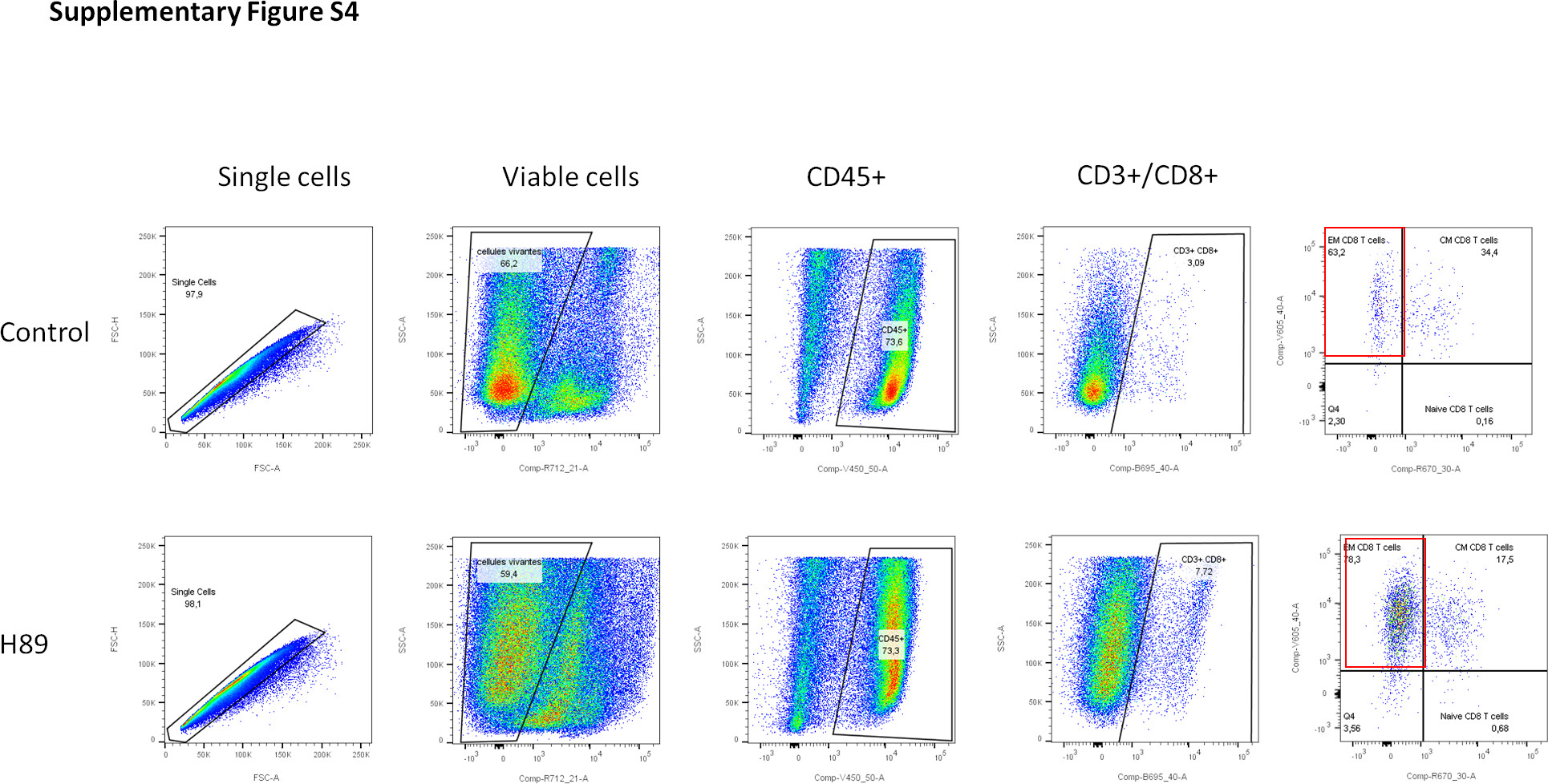

Supplement: Supplementary Figure 4 — Flow cytometry dot plot representation of effector memory CD8+ T cells at D14 after H89 treatment. Representation of the gating strategy on CT26 tumors treated with H89 at D14 (10 mg/kg, i.p., NaCl in the control group) for the identification of effector memory (EM) CD8 T cells. EM CD8 T cells are highlighted in the red frame. [file Image_4.jpeg]

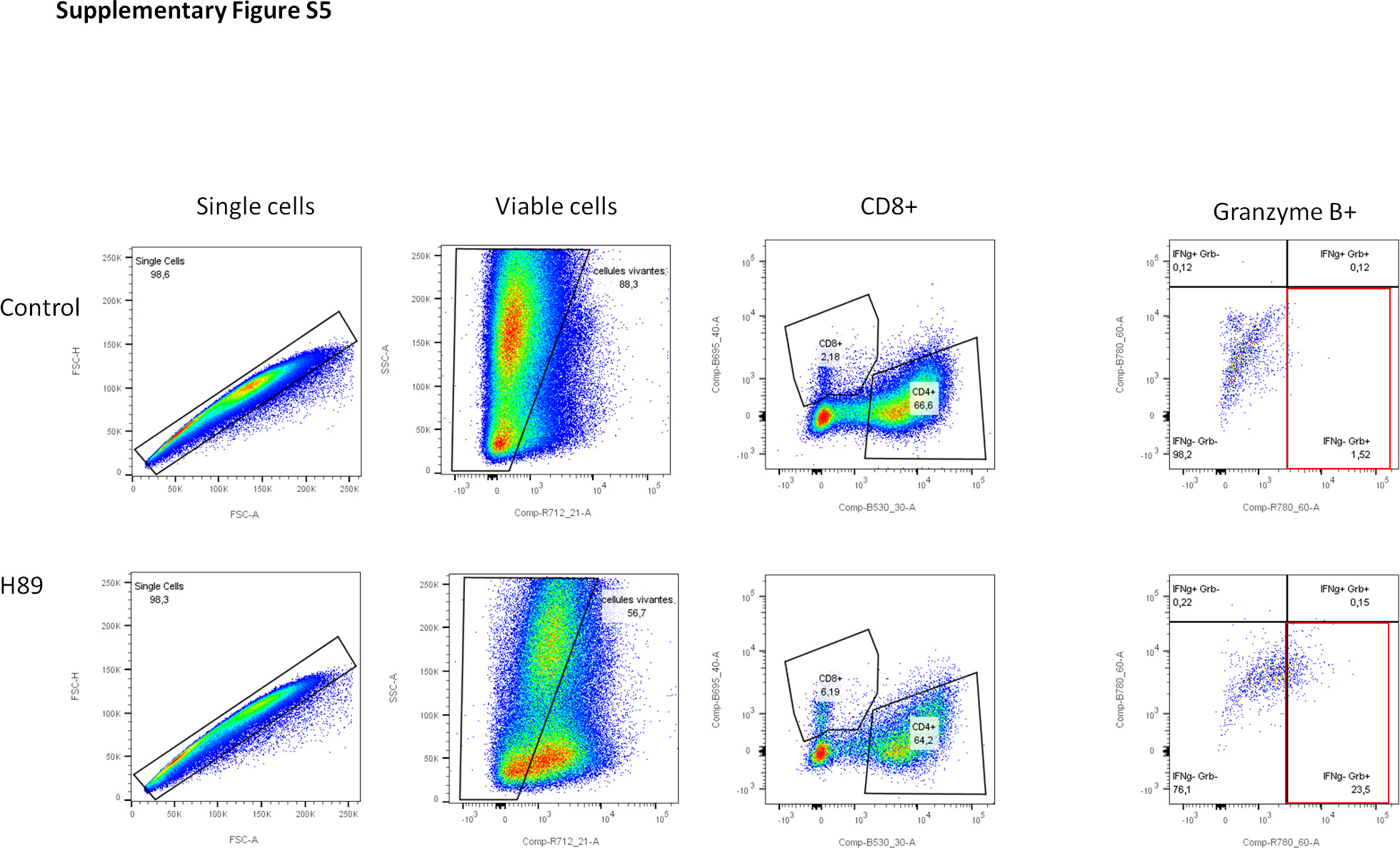

Supplement: Supplementary Figure 5 — Flow cytometry dot plot representation of CD8+/GranzymeB+ CD8 T cells at D14 after H89 treatment. Representation of the gating strategy on CT26 tumors treated with H89 at D14 (10 mg/kg, i.p., NaCl in the control group) for the identification of Granzyme B+ CD8 T cells which are highlighted in the red frame. [file Image_5.jpeg]

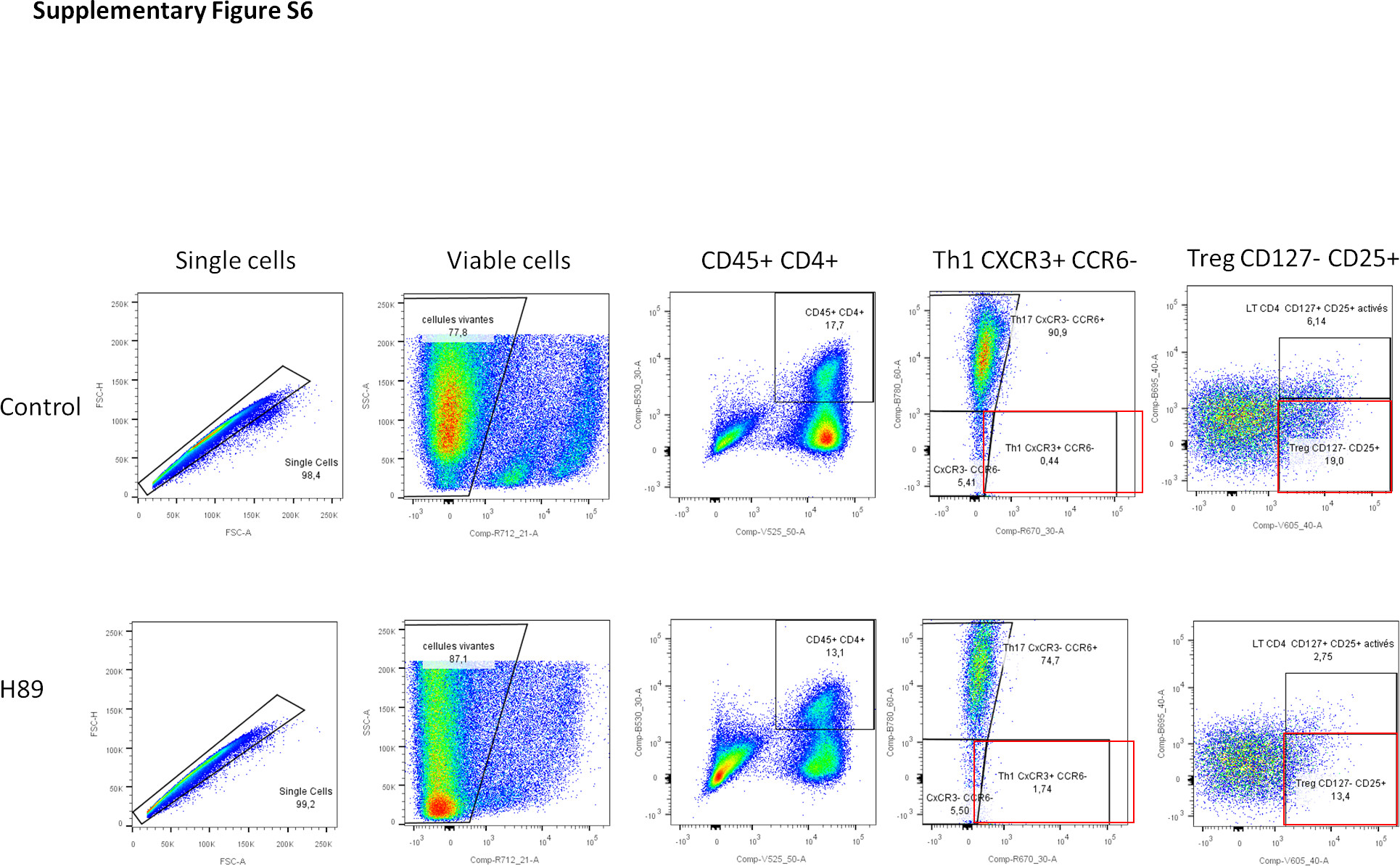

Supplement: Supplementary Figure 6 — Flow cytometry dot plot representation of Th1 and Treg cells at D14 after H89 treatment. Representation of the gating strategy on CT26 tumors treated with H89 at D14 (10 mg/kg, i.p., NaCl in the control group) for the identification of Th1 and Treg cells which are highlighted in the red frame. [file Image_6.jpeg]
