# Supplementary material for: Protein Kinase Inhibitor-Mediated Immunoprophylactic and Immunotherapeutic Control of Colon Cancer
Source: Front Immunol. 2022 Apr 28;13:875764. doi: 10.3389/fimmu.2022.875764 (PMC9097540; doi:10.3389/fimmu.2022.875764)
Supplement: Supplementary file 7 [file Table_1.pdf]

Supplementary table S1. RT-qPCR primers

Primers (mouse)	Forward	Reverse
Cxcl10	AGA ACG GTG CGC TGC AC	CCT ATG GCC CTG GGT CTC A
IFNγ	GAGCTCATTGAATGCTTGGC	GCGTCATTGAATCACACCTG
Tbet	GCCAGGGAACCGCTTATATG	GACGATCATCTGGGTCACATTGT
FoxP3	CCTATGGCTCCTTCCTTGGC	ATGAAGTGTGGTCTGTCCTGG
IL-15	CATATGGAATCCAACTGGATAGATGTAAGATA	CATATGCTCGAGGGACGTGTTGATGAACAT
Actine	GGCACCACACCTTCTACAATGAGC	CGACCAGAGGCATACAGGGACAG
