# Supplementary material for: Protein Kinase Inhibitor-Mediated Immunoprophylactic and Immunotherapeutic Control of Colon Cancer
Source: Front Immunol. 2022 Apr 28;13:875764. doi: 10.3389/fimmu.2022.875764 (PMC9097540; doi:10.3389/fimmu.2022.875764)
Supplement: Supplementary file 8 [file Table_2.pdf]

Supplementary table S2. Flow cytometry antibodies for CD4+ TLs populations characterisation

Antibodies	References
CD45 VioGreen (clone REA737)	Miltenyi (130-110-803)
CD4 FITC (clone RM4-4)	BD Pharmingen™ (553055)
CD25 BV605 (clone PC61)	BioLegend (102035)
CD127 PerCP/Cy5.5 (clone A7R34)	BioLegend (135021)
CxCr5 BV421 (clone L138D7)	BioLegend (145511)
ST2(IL-33R) PE (clone DJ8)	Mdbioproducts (101001PE)
CCR6 PE-Vio (clone REA277)	Miltenyi (130-103-818)
CxCr3 APC (clone REA724)	Miltenyi (130-111-088)
PD-1 APC/Cy7 (clone 29F.1A12)	BioLegend (135223)
