# Supplementary material for: Protein Kinase Inhibitor-Mediated Immunoprophylactic and Immunotherapeutic Control of Colon Cancer
Source: Front Immunol. 2022 Apr 28;13:875764. doi: 10.3389/fimmu.2022.875764 (PMC9097540; doi:10.3389/fimmu.2022.875764)
Supplement: Supplementary file 9 [file Table_3.pdf]

Supplementary table S3. Flow cytometry antibodies for $CD8^+$ T cells characterisation

Antibodies	References
CD45 Pacific Blue (clone 30F11)	BioLegend (103126)
CD44 BV 605 (clone IM7)	BioLegend (103047)
CD3 FITC (clone 145-2C11)	BioLegend (100306)
CD8a PerCP/Cy5.5 (clone 53-6.7)	BioLegend (100734)
CD62L APC (clone MEL-14)	BioLegend (104411)
