# Supplementary material for: Protein Kinase Inhibitor-Mediated Immunoprophylactic and Immunotherapeutic Control of Colon Cancer
Source: Front Immunol. 2022 Apr 28;13:875764. doi: 10.3389/fimmu.2022.875764 (PMC9097540; doi:10.3389/fimmu.2022.875764)
Supplement: Supplementary file 10 [file Table_4.pdf]

Supplementary table S4. Flow cytometry antibodies for CD8^{+} and CD4^{+} T cells intracellular staining.

Antibodies	References
CD45 VioGreen (clone REA737)	Miltenyi (130-110-803)
CD4 FITC (clone RM4-4)	BD Pharmingen™ (553055)
CD8a PerCP/Cy5.5 (clone 53-6.7)	BioLegend (100734)
IFNγ PE/Cy7 (clone XMG1.2)	BD Biosciences (557649)
Granzyme B APC-Fire750 (clone QA16A02)	BioLegend (372210)
