# Supplementary material for: Protein Kinase Inhibitor-Mediated Immunoprophylactic and Immunotherapeutic Control of Colon Cancer
Source: Front Immunol. 2022 Apr 28;13:875764. doi: 10.3389/fimmu.2022.875764 (PMC9097540; doi:10.3389/fimmu.2022.875764)
Supplement: Supplementary file 11 [file Table_5.pdf]

*Supplementary table S5. Flow cytometry antibodies for PD-1, PD-L1 and CD80 labeling*

| Antibodies | References |
|---|---|
| PD-1 Pacific Blue (clone EH12.2H7) | BioLegend (329915) |
| PD-1 APC/Cy7 (clone 29F.1A12) | BioLegend (135223) |
| PD-L1 APC (clone 10F9G2) | Invitrogen (A14929) |
| CD80 FITC (clone 16-10A1) | BioLegend (104706) |
